# Ezetimibe combination therapy with statin for non-alcoholic fatty liver disease: an open-label randomized controlled trial (ESSENTIAL study)

**DOI:** 10.1186/s12916-022-02288-2

**Published:** 2022-03-21

**Authors:** Yongin Cho, Hyungjin Rhee, Young-eun Kim, Minyoung Lee, Byung-Wan Lee, Eun Seok Kang, Bong-Soo Cha, Jin-Young Choi, Yong-ho Lee

**Affiliations:** 1 Department of Endocrinology and Metabolism, Inha University School of Medicine, Incheon, Republic of Korea; 2 Graduate School, Yonsei University College of Medicine, Seoul, Republic of Korea; 3 Department of Radiology, Research Institute of Radiological Science, Severance Hospital, Yonsei University College of Medicine, Seoul, Republic of Korea; 4 Department of Internal Medicine, Yonsei University College of Medicine, 50-1, Yonsei-ro, Seodaemun-gu, Seoul, 03722 Republic of Korea; 5 Institute of Endocrine Research, Yonsei University College of Medicine, Seoul, Republic of Korea; 6 Department of Systems Biology, Glycosylation Network Research Center, Yonsei University, Seoul, Republic of Korea

**Keywords:** Niemann-Pick C1-like 1 inhibitor, Statins, Hepatic steatosis, Hepatic fibrosis

## Abstract

**Background:**

The effect of ezetimibe, Niemann-Pick C1-like 1 inhibitor, on liver fat is not clearly elucidated. Our primary objective was to evaluate the efficacy of ezetimibe plus rosuvastatin versus rosuvastatin monotherapy to reduce liver fat using magnetic resonance imaging-derived proton density fat fraction (MRI-PDFF) in patients with non-alcoholic fatty liver disease (NAFLD).

**Methods:**

A randomized controlled, open-label trial of 70 participants with NAFLD confirmed by ultrasound who were assigned to receive either ezetimibe 10 mg plus rosuvastatin 5 mg daily or rosuvastatin 5 mg for up to 24 weeks. The liver fat change was measured as average values in each of nine liver segments by MRI-PDFF. Magnetic resonance elastography (MRE) was used to measure liver fibrosis change.

**Results:**

Combination therapy significantly reduced liver fat compared with monotherapy by MRI-PDFF (mean difference: 3.2%; *p* = 0.020). There were significant reductions from baseline to study completion by MRI-PDFF for both the combination and monotherapy groups, respectively (18.1 to 12.3%; *p* < 0.001 and 15.0 to 12.4%; *p* = 0.003). Individuals with higher body mass index, type 2 diabetes, insulin resistance, and severe liver fibrosis were likely to be good responders to treatment with ezetimibe. MRE-derived change in liver fibrosis was not significantly different (both groups, *p* > 0.05). Controlled attenuation parameter (CAP) by transient elastography was significantly reduced in the combination group (321 to 287 dB/m; *p* = 0.018), but not in the monotherapy group (323 to 311 dB/m; *p* = 0.104).

**Conclusions:**

Ezetimibe and rosuvastatin were found to be safe to treat participants with NAFLD. Furthermore, ezetimibe combined with rosuvastatin significantly reduced liver fat in this population.

**Trial registration:**

The trial was registered at ClinicalTrials.gov (registration number: NCT03434613).

**Supplementary Information:**

The online version contains supplementary material available at 10.1186/s12916-022-02288-2.

## Background

The incidence of non-alcoholic fatty liver disease (NAFLD) is increasing globally and is closely related to metabolic syndrome features including obesity, insulin resistance, dyslipidemia, and diabetes mellitus (DM). Cardiovascular disease, which is closely related to dyslipidemia, is a major cause of mortality in patients with NAFLD [1]. Furthermore, NAFLD independently increases the risk of cardiovascular complications [2]. Therefore, management of dyslipidemia in patients with NAFLD is an important concern. In recent treatment guidelines for dyslipidemia, more aggressive treatment to target low-density lipoprotein cholesterol (LDL-C) is recommended [3]. Statins, the most potent drugs that lower LDL-C, are known to have several side effects whose risks appear to increase with higher doses [4]. For this reason, concomitant use of second-line drugs such as ezetimibe with a lower statin dose may be beneficial.

Cholesterol alone can contribute to the progression of non-alcoholic steatohepatitis (NASH) [5], and the effects of anti-dyslipidemia therapies on hepatic steatosis and/or fibrosis have been investigated. Evidence of benefit by using a statin in patients with NAFLD is limited [6], and statin was not beneficial in improving NASH in a previous randomized controlled trial [7]. Also, the effect of ezetimibe on hepatic steatosis is still controversial. Ezetimibe exhibits lipid-lowering effects through inhibition of Niemann-Pick C1-like 1 (NPC1-L1) in the intestine and has been reported to reduce visceral fat and improve insulin resistance in several studies [8, 9]. Improvement of hyperinsulinemia may lead to the inhibition of sterol regulatory element-binding protein-1c (SREBP-1c) and blockade of fatty acid synthase [10]. And this mechanism can contribute to improvement in hepatic steatosis. Recently, our group reported that the use of ezetimibe affects autophagy of hepatocytes and improves hepatic steatosis in an animal model [11].

However, the effects of ezetimibe have not been clearly elucidated in human studies. Ezetimibe did not significantly reduce liver fat in a previous randomized controlled trial by Loomba et al. [12]. This study was designed using ezetimibe alone, with the use of concomitant statin drugs not clearly controlled. Considering that the improvement of cardiovascular disease risk through ezetimibe has been identified in combination with statins [13], increased understanding of ezetimibe effect on NAFLD in combination with a statin will be of great clinical significance. Therefore, in this study, we investigated whether ezetimibe contributes to hepatic steatosis improvement in the setting of controlled statin treatment.

## Methods

### Study design and patient population

The ESSENTIAL (Effects of Statin Monotherapy and Statin/Ezetimibe Combination Therapy on Non-alcoholic Steatohepatitis in patients with Hyperlipidemia and Fatty Liver) study was an investigator-initiated, randomized, open-label, prospective, active-controlled clinical trial to examine the efficacy of ezetimibe 10 mg/day orally combined with rosuvastatin 5 mg/day versus rosuvastatin 5 mg/day orally for up to 24 weeks to improve hepatic steatosis as measured by magnetic resonance imaging-derived proton density fat fraction (MRI-PDFF). Participants of the study were recruited from Severance Hospital from May 2018 to June 2019 in Seoul, Korea.

The trial was registered at ClinicalTrials.gov (registration number: NCT03434613). All participants provided written informed consent and the Ethics Committee of the Yonsei University College of Medicine approved this study (4-2017-1168), which conforms to the ethical principles of the 1975 Declaration of Helsinki.

### Inclusion criteria

Patients were required to meet all the following criteria for study inclusion: 19–80 years of age, hepatic steatosis documented by abdominal ultrasound, hyperlipidemia indicated to treat by the domestic dyslipidemia treatment guideline [14], diagnosed with type 2 diabetes (T2DM, HbA1c ≤9.0% with no change in type of oral or injectable hypoglycemic agent/s within 12 weeks of enrollment) or without diabetes, and written informed consent. Exclusion criteria are provided in the Supplementary Appendix (Additional file 1).

### Baseline assessment at screening

All patients underwent a baseline assessment before undergoing randomization, including medical history and physical exam. For those who had undergone abdominal ultrasonography within the past 1 year, test results were reviewed for the presence of hepatic steatosis. Otherwise, abdominal ultrasonography was performed for screening purposes.

### Randomization

A random allocation sequence was computer-generated elsewhere, and participants were assigned in a 1:1 ratio to receive treatment with ezetimibe plus rosuvastatin or rosuvastatin alone. To equalize the proportion of type 2 diabetes in both groups, patients were stratified according to the presence of diabetes before randomization.

### Primary and secondary outcomes

The primary outcome was change in liver fat by MRI-PDFF quantified as the average in colocalized regions of interest (ROI) within each of nine liver segments [15]. Secondary outcomes were change in liver fibrosis by magnetic resonance elastography (MRE), change in insulin sensitivity determined by homeostatic model assessment insulin resistance (HOMA-IR), and changes in parameters including body weight, body mass index (BMI), waist circumference (WC), systolic and diastolic blood pressure, fasting glucose, HbA1c, free fatty acids, platelets, alkaline phosphatase, total bilirubin, aspartate aminotransferase (AST), alanine aminotransferase (ALT), total cholesterol, triglyceride, high-density lipoprotein cholesterol (HDL-C), LDL-C, high sensitivity C-reactive protein, HOMA-beta, and biomarkers including interleukin (IL)-1 beta, IL-8, and IL-18. Transient elastography (TE) (Fibroscan®; Echosens, Paris, France) was also performed before and after treatment, and changes in the hepatic steatosis index-controlled attenuation parameter (CAP) and hepatic fibrosis index-liver stiffness measurement (LSM) were also included as secondary outcome measures.

### MRI-PDFF for fat quantification and MRE for liver fibrosis quantification

For hepatic fat quantification, MRI-PDFF sequence (mDIXON Quant) was used, and a fat fraction map was automatically generated by the manufacturer’s console. For liver fibrosis measurement, MRE was performed using a 2-dimensional gradient-echo sequence and a passive driver placed on the participant’s right upper abdomen (see Additional file 1: Supplementary Appendix for details and Table S1).

### Statistical analysis

All categorical variables were expressed as number (proportion) and compared by *χ*^2^ analysis. The independent samples *t*-test or Wilcoxon-Mann-Whitney *U* test was used for differences between continuous variables. For the primary objective, per-protocol analysis was performed. Wilcoxon signed-rank test or logistic regression was used for additional analysis, when indicated.

We expected the ezetimibe treatment group to have a liver fat reduction of >10% compared to baseline and the statin monotherapy group to have a <5% reduction compared to baseline. We also expected a dropout rate of 15–20%; thus, we planned to enroll 70 participants to achieve power of at least 80% with *α* and 0.05 with *β* assuming study completion by ≥29 participants per arm.

Additional analyses for treatment response were conducted across multiple subgroups to examine whether the effect of ezetimibe differed on the basis of patient demographics or clinical characteristics. Characteristics for subgroup analysis included age (age 50 or more, under age 50), sex (men, women), body mass index (30 kg/m^2^ or more, under 30 kg/m^2^), presence of type 2 diabetes, sarcopenia (defined as a skeletal muscle mass index (SMI, %) <2 standard deviations below the gender-specific mean for the Korean population: SMI (%) <29.0 in men and <22.9 in women) [16], HOMA-IR (over median value of the whole study population, under median value), baseline MRI-PDFF (over median value, under median value), and baseline MRE (over median value, under median value). Response was defined as ≥30% relative reduction or ≥5% absolute decline in MRI-PDFF from baseline to end of treatment [17].

All statistical analyses were performed using IBM SPSS Statistics version 26.0 (IBM Corp., Armonk, NY, USA).

## Results

### Baseline characteristics of study participants

**Fig. 1:**
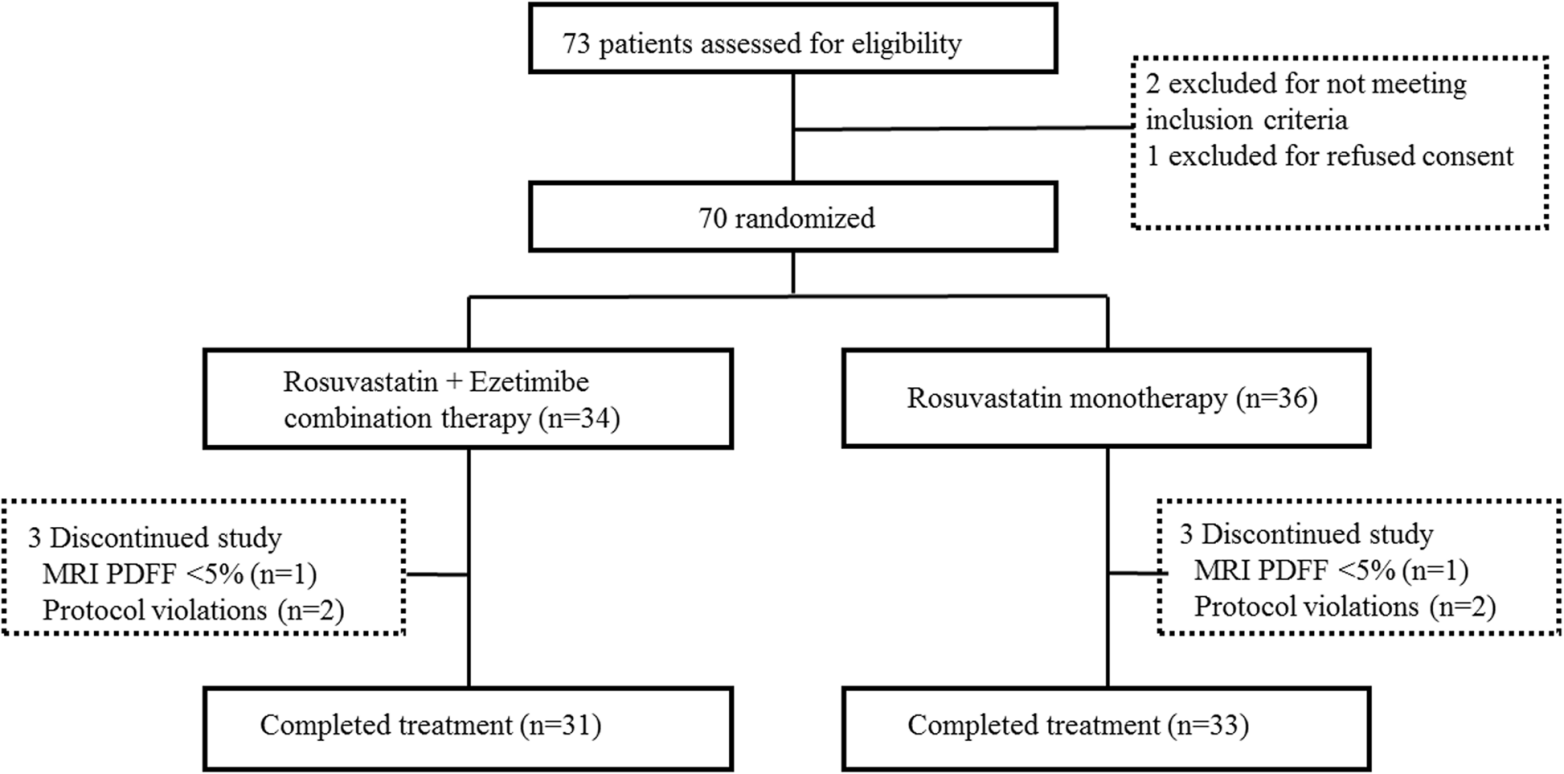
CONSORT flow diagram. Abbreviations: CONSORT, Consolidated Standard of Reporting Trial

A total of 70 participants with NAFLD were randomly assigned to receive either combination therapy with ezetimibe plus rosuvastatin (*n* = 34) or rosuvastatin monotherapy (*n* = 36). Three subjects in the combination group and 3 patients in the monotherapy group were excluded from the final analysis (Fig. 1). Baseline demographic and biochemical profiles are summarized in Table 1, showing that both groups had comparable characteristics at study entry. The study population included 42.9% women and 74.3% of participants had type 2 diabetes. All participants were Asian with an average BMI of 28.5±3.5 kg/m^2^. The mean PDFF value before treatment was 16.5%±7.8% and the mean CAP value was 313.7 dB/m±33.2 dB/m for the whole study population.

**Table 1:** Baseline demographic and biochemical characteristics

	Ezetimibe + rosuvastatin (*n* = 34)	Rosuvastatin alone (*n* = 36)	*p*-value
Demographics			
Age, years	50.3 (12.9)	52.5 (19.1)	0.647
Female; *n* (%)	12 (35.3)	18 (50.0)	0.214
Weight, kg	78.2 (19.3)	77.2 (15.5)	0.958
BMI, kg/m^2^	27.7 (6.6)	28.6 (3.6)	0.196
Waist circumference, cm	95.0 (15.3)	96.5 (11.1)	0.685
Presence of diabetes, *n* (%)	26 (72.2)	26 (76.5)	0.684
Seated SBP, mmHg	126.0 (11.0)	123.0 (13.0)	0.676
Seated DBP, mmHg	81.0 (11.0)	80.5 (13.0)	0.851
Biochemical profile			
ALT, IU/l	37.5 (30.0)	30.0 (22.0)	0.099
AST, IU/l	24.5 (13.0)	24.5 (13.0)	0.706
Platelets, ×10^3^/μl	263.0 (89.0)	258.5 (77.0)	0.307
Alk Phos, U/l	64.0 (22.0)	68.5 (36.0)	0.121
GGT, U/l	42.5 (36.0)	35.0 (33.0)	0.347
Total bilirubin, mg/dl	0.6 (0.3)	0.7 (0.4)	0.557
Glucose, mg/dl	116.0 (26.0)	114.0 (35.0)	0.958
Insulin, uU/ml	12.4 (10.3)	14.7 (9.1)	0.171
HbA1c, %	6.4 (0.5)	6.4 (0.9)	0.548
FFA, mmol/l	509.0 (307.0)	532.5 (260.0)	0.541
HOMA-IR	3.5 (3.5)	3.9 (2.7)	0.166
HOMA-beta	85.5 (70.3)	99.6 (60.4)	0.347
Triglycerides, mg/dl	176.5 (143.0)	217.5 (159.0)	0.177
HDL, mg/dl	45.0 (16.0)	42.0 (8.0)	0.266
LDL, mg/dl	115.3 (64.7)	111.2 (39.9)	0.742
CRP, mg/l	1.5 (1.8)	1.5 (1.5)	0.897
eGFR, CKD-EPI equation	102.0 (23.0)	95.5 (28.0)	0.347

Data are expressed as median (IQR) unless indicated as number (%) with *p*-values from the Mann-Whitney *U* test or *χ*^2^ analysis

*Abbreviations*: *BMI* body mass index, *SBP* systolic blood pressure, *DBP* diastolic blood pressure, *ALT* alanine aminotransferase, *AST* aspartate aminotransferase, *GGT* gamma-glutamyl transferase, *FFA* free fatty acids, *HOMA-IR* homeostatic model assessment of insulin resistance, *HDL* high-density lipoprotein, *LDL* low-density lipoprotein, *CRP* C-reactive protein, *eGFR* estimated glomerular filtration rate

### Effect of rosuvastatin with/without ezetimibe on liver fat as assessed by MRI-PDFF

**Fig. 2:**
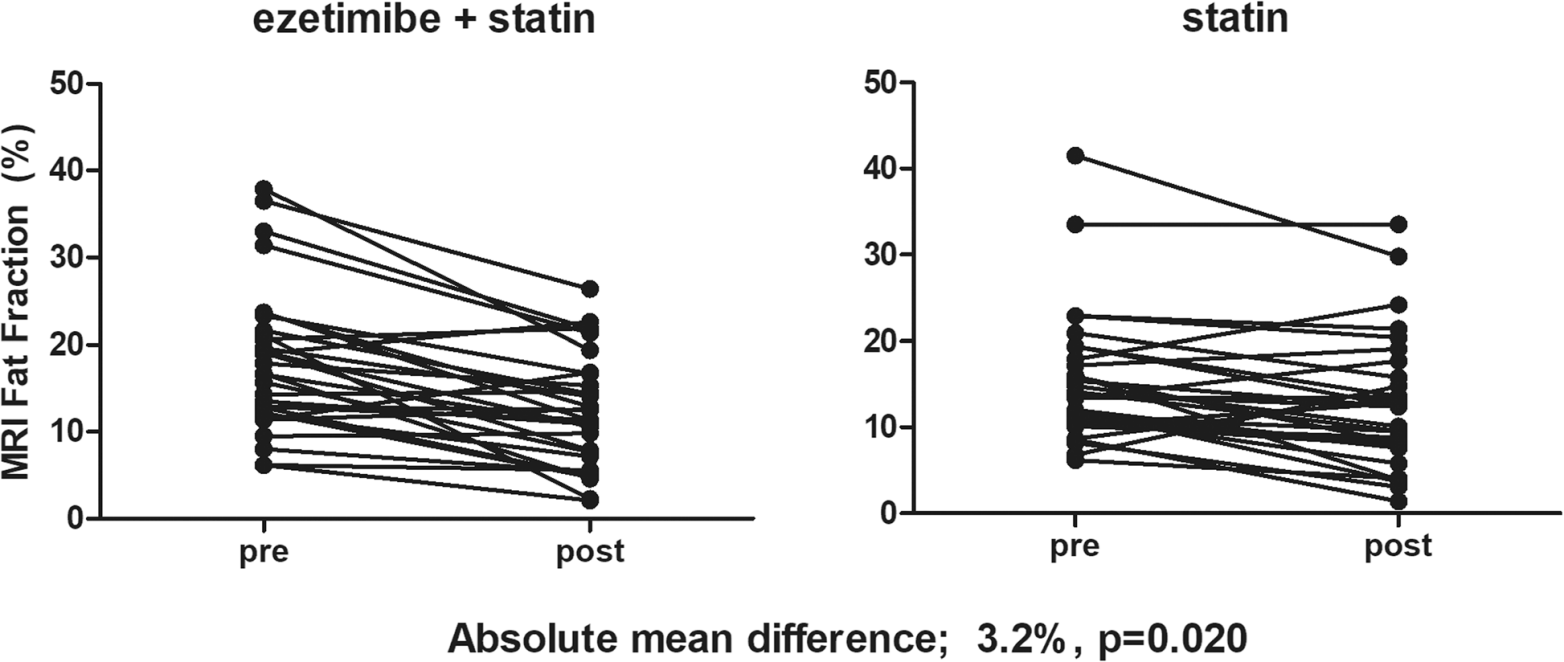
Liver fat (%) by MRI-PDFF at baseline and end of treatment by regimen

Compared to baseline, there were significant reductions in end-of-treatment MRI-PDFF for both the combination group (18.1 to 12.3%; *p* < 0.001) and the monotherapy group (15.0 to 12.4%; *p* = 0.003) (Table 2 and Additional file 1: Fig. S1A). In the per-protocol analysis, ezetimibe combined with rosuvastatin was significantly better than rosuvastatin monotherapy in reducing liver fat measured by MRI-PDFF (mean difference 3.2%; *p* = 0.020) (Fig. 2). The degree to which hepatic steatosis improved was similar among individual segments within the treatment group regardless of treatment assignment.

**Table 2:** Ezetimibe plus rosuvastatin versus rosuvastatin monotherapy: longitudinal changes in liver fat and liver fibrosis

		Ezetimibe + rosuvastatin (*n* = 31)			Rosuvastatin alone (*n* = 33)		Difference between groups
Liver segment	Baseline	Post-treatment	*p*-value	Baseline	Post-treatment	*p*-value	*p*-value
I	16.6 (8.2)	11.3 (6.4)	< 0.001	13.2 (7.3)	11.5 (7.0)	0.042	0.012
II	16.7 (8.4)	10.8 (6.0)	< 0.001	14.2 (7.4)	11.8 (7.4)	0.006	0.012
III	17.1 (8.3)	11.2 (6.6)	< 0.001	14.2 (7.6)	11.9 (7.2)	0.012	0.011
IV (A)	18.0 (8.4)	12.4 (6.7)	< 0.001	15.3 (7.9)	12.5 (7.5)	0.004	0.038
IV (B)	18.9 (8.8)	13.4 (7.5)	< 0.001	15.9 (7.3)	13.2 (7.8)	0.003	0.051
V	18.9 (8.7)	12.9 (6.7)	< 0.001	16.1 (7.6)	13.2 (7.8)	0.002	0.029
VI	18.1 (8.3)	12.4 (6.7)	< 0.001	14.5 (7.5)	11.7 (7.6)	0.001	0.039
VII	18.6 (7.9)	12.5 (6.5)	< 0.001	15.2 (7.2)	12.4 (7.3)	0.001	0.018
VIII	19.6 (8.5)	13.5 (7.2)	< 0.001	16.6 (7.4)	13.6 (7.9)	0.002	0.028
MRI-PDFF average, %	18.1 (8.2)	12.3 (6.4)	< 0.001	15.0 (7.3)	12.4 (7.4)	0.003	0.020
MRE, kPa	2.0 (0.5)	2.1 (0.5)	0.507	2.2 (0.4)	2.2 (0.7)	0.539	0.898

Magnetic resonance imaging proton density fat fraction (MRI-PDFF) with colocalized MRI measurements was used in the longitudinal liver fat mapping. Longitudinal changes in liver fibrosis were measured using magnetic resonance elastography (MRE). Data are expressed as mean (SD) or difference with *p*-values from paired *t*-test or per-protocol analysis

*Abbreviations*: *MRI-PDFF* magnetic resonance imaging proton density fat fraction, *MRE* magnetic resonance elastography

### Effect of rosuvastatin with/without ezetimibe on liver fibrosis as assessed by MRE

Compared to baseline, there were no significant differences at end of treatment by MRE in either the combination group or monotherapy group (2.0 to 2.1 kPa; *p* = 0.507 and 2.2 to 2.2 kPa; *p* = 0.539, respectively) (Table 2 and Additional file 1: Fig. S1B). MRE-derived liver fibrosis was not significantly different between groups (*p* = 0.898).

### Effect of rosuvastatin with/without ezetimibe on liver fat and fibrosis as assessed by transient elastography

We also evaluated changes in liver fat and fibrosis by TE. A significant decrease in CAP at end of treatment was observed for ezetimibe plus rosuvastatin (321 to 287 dB/m; *p*=0.018), but not for the rosuvastatin alone group (323 to 311 dB/m; *p*=0.104, Additional file 1: Table S2). However, the between-group difference was not statistically significant (*p*=0.253). In terms of hepatic fibrosis, there was no significant difference in LSM from baseline to end of treatment in the combination group (6.0 to 6.1 kPa; *p*=0.339) or the monotherapy group (6.7 to 6.0 kPa; *p*=0.881).

### Effect of rosuvastatin with/without ezetimibe on biochemical indices

Changes in anthropometric and biochemical variables are summarized in Table 3. There were significant decreases in BMI, waist circumference, triglyceride, LDL cholesterol, and C-reactive protein (CRP) in both groups (all *p*<0.05). However, changes in all variables including BMI, alanine aminotransferase, aspartate aminotransferase, fasting glucose, HbA1c, and homeostatic model assessment insulin resistance were not significantly different between the two groups (all *p* > 0.05).

**Table 3:** Changes in parameters after 24 weeks of treatment with ezetimibe plus rosuvastatin versus rosuvastatin monotherapy

		Ezetimibe + rosuvastatin (*n*=31)			Rosuvastatin alone (*n*=33)		Difference between groups
	Baseline	Post-treatment	*p*-value	Baseline	Post-treatment	*p*-value	*p*-value
BMI, kg/m^2^	26.6 (6.4)	26.2 (5.8)	0.002	28.3 (3.6)	27.9 (3.7)	0.001	0.675
Waist circumference, cm	94.0 (15.0)	91.0 (13.0)	<0.001	96.0 (12.5)	93.5 (14.0)	<0.001	0.269
Seated SBP, mmHg	127.0 (10.4)	127.2 (12.7)	0.936	128.3 (13.0)	126.2 (11.5)	0.341	0.450
Seated DBP, mmHg	81.5 (7.3)	80.0 (10.1)	0.409	82.0 (9.8)	78.2 (8.7)	0.020	0.354
ALT, IU/l	40.0 (31.0)	40.0 (24.0)	0.459	31.0 (21.0)	32.0 (26.0)	0.563	0.471
AST, IU/l	25.0 (14.0)	26.0 (15.0)	0.681	24.0 (11.0)	27.0 (18.0)	0.727	0.462
Platelets, ×10^3^/μl	265.0 (87.0)	259.0 (82.0)	0.175	255.0 (80.0)	248.0 (75.5)	0.003	0.357
Alk Phos, U/l	64.0 (21.0)	63.0 (18.0)	0.642	71.0 (36.0)	70.0 (32.5)	0.326	0.510
GGT, U/l	43.0 (35.0)	32.0 (41.0)	0.125	36.0 (33.5)	33.0 (27.5)	0.957	0.861
Total bilirubin, mg/dl	0.6 (0.3)	0.6 (0.4)	0.871	0.7 (0.4)	0.7 (0.4)	0.165	0.600
Glucose, mg/dl	116.0 (29.0)	116.0 (40.0)	0.378	115.0 (33.5)	120.0 (25.0)	0.957	0.345
Insulin, uU/ml	12.5 (9.3)	12.7 (6.9)	0.931	15.0 (8.0)	15.5 (12.1)	0.993	0.911
HbA1c, %	6.4 (0.6)	6.5 (0.8)	0.167	6.5 (1.1)	6.5 (1.4)	0.055	0.445
FFA, mmol/l	480.0 (314.0)	506.0 (318.0)	0.433	515.0 (279.5)	403.0 (316.5)	0.059	0.545
HOMA-IR	3.6 (3.7)	3.5 (3.0)	0.814	4.0 (3.5)	4.7 (3.3)	0.925	0.691
HOMA-beta	87.0 (65.6)	91.7 (81.2)	0.299	99.6 (59.6)	94.0 (81.6)	0.957	0.098
Triglycerides, mg/dl	177.0 (139.0)	138.0 (77.0)	<0.001	217.0 (157.0)	135.0 (71.0)	<0.001	0.175
HDL, mg/dl	45.0 (16.0)	44.0 (10.0)	0.045	42.0 (7.5)	41.0 (11.0)	0.948	0.109
LDL, mg/dl	116.0 (66.4)	55.0 (37.2)	<0.001	109.8 (42.9)	66.4 (28.3)	<0.001	0.111
CRP, mg/l	1.4 (1.7)	0.8 (1.2)	0.036	1.5 (1.4)	0.8 (1.6)	0.008	0.805
tPAI-1, ng/ml	17.8 (10.2)	12.5 (17.3)	0.116	21.7 (14.4)	17.5 (12.2)	0.016	0.217
IL-1 β, pg/ml	0.045 (0.085)	0.077 (0.111)	0.144	0.063 (0.082)	0.076 (0.124)	0.296	0.843
IL-8, pg/ml	3.3 (2.5)	2.6 (2.1)	0.122	3.7 (2.3)	2.9 (2.1)	0.088	0.866
IL-18, pg/ml	163.6 (73.8)	146.2 (52.0)	0.003	168.2 (73.8)	162.6 (76.8)	0.042	0.210

Data are expressed as median (IQR) with *p*-values from Wilcoxon signed-rank test. *p*-value difference was determined using per-protocol analysis

*Abbreviations*: *BMI* body mass index, *SBP* systolic blood pressure, *DBP* diastolic blood pressure, *ALT* alanine aminotransferase, *AST* aspartate aminotransferase, *GGT* gamma-glutamyl transferase, *FFA* free fatty acids, *HOMA-IR* homeostatic model assessment of insulin resistance, *HDL* high-density lipoprotein, *LDL* low-density lipoprotein, *CRP* C-reactive protein, *tPAI* total plasminogen activator inhibitor, *IL* interleukin

To explore drug mechanisms, additional tests were performed on several values known to be inflammation markers of NASH progression. Significant improvement was reported in IL-18 in the combination group and in tPAI-1 and IL-18 in the monotherapy group, but there was no significant difference in treatment effect between groups (all *p* > 0.05).

### Prediction of response by ezetimibe combination treatment

**Fig. 3:**
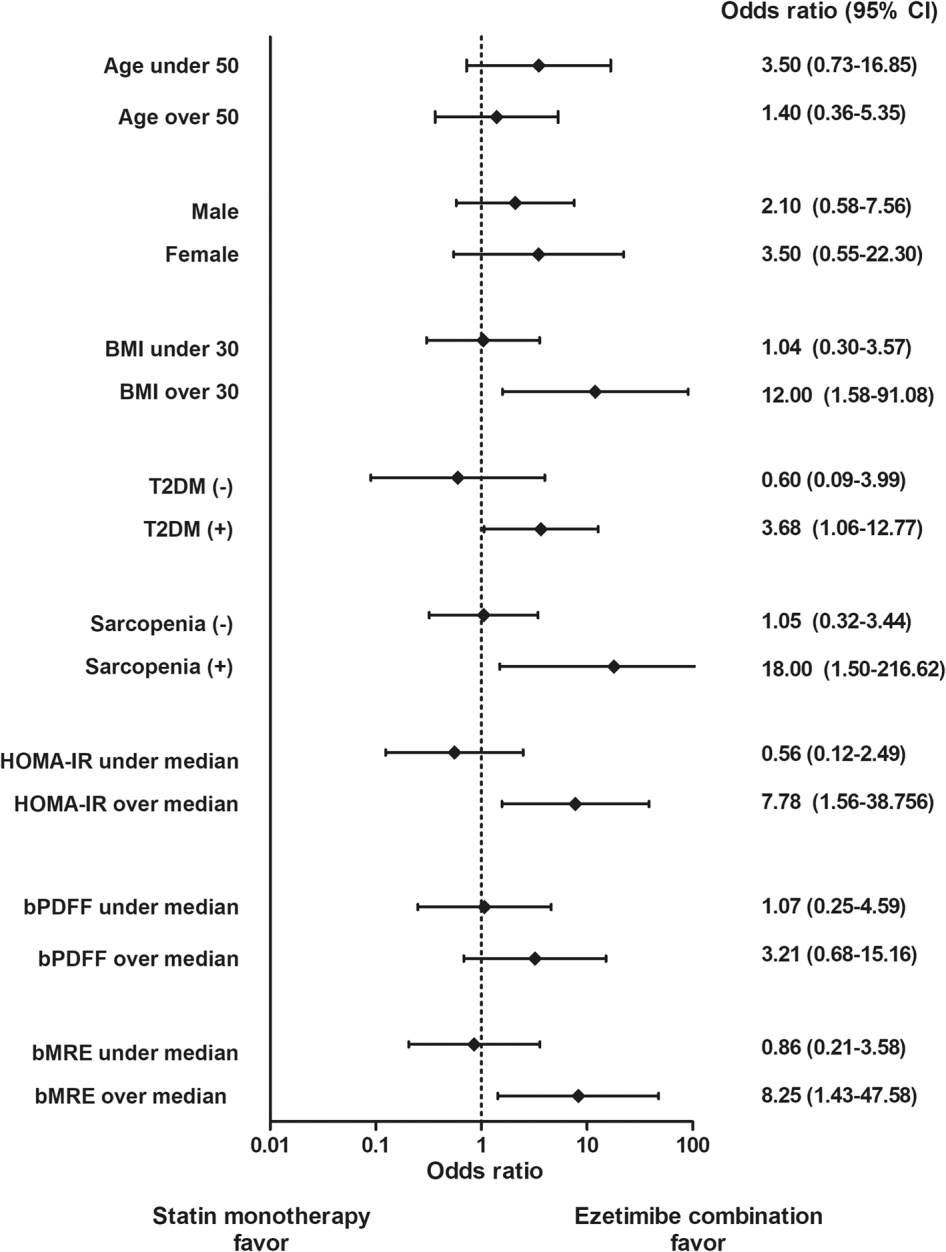
Odds ratio of steatosis improvement by subgroups. Response (≥30% relative reduction or ≥5% absolute decline by MRI-PDFF from baseline to end of treatment) by age (≥ or <50), sex, body mass index (≥ or < 30 kg/m^2^), type 2 diabetes, sarcopenia (skeletal muscle mass index % <2 standard deviations below gender-specific mean for the Korean population: men, 29.0; women, 22.9), HOMA-IR (≥3.7, study median), baseline MRI-PDFF (≥14.3%, study median), and baseline MRE (≥2.1, study median)

Defining response as a ≥30% relative reduction or ≥5% absolute decline in MRI-PDFF from baseline to end of treatment, ezetimibe combination treatment more effectively improved hepatic steatosis in participants with higher BMI (≥30 kg/m^2^), presence of T2DM, presence of sarcopenia, increased HOMA-IR (≥3.7, the median value of the study population), and increased baseline MRE (≥2.1, the median value in this study) (Fig. 3).

### Adverse events

Three participants in each treatment arm dropped out of the study, but this was not associated with adverse events. One subject in each group had no documented hepatic steatosis (<5% on MRI-PDFF) at baseline. Two participants in each group were discontinued due to protocol violations. There was no significant adverse event reported in this study.

## Discussion

This randomized controlled clinical trial showed ezetimibe with rosuvastatin was significantly better than rosuvastatin monotherapy for reducing liver fat as measured by MRI-PDFF. Consistent with this finding, a significant reduction of liver fat was also confirmed in end-of-treatment CAP measured by TE, but only for the combination group. Ezetimibe combination treatment did not significantly decrease fibrosis as measured by MRE or TE. In subgroup analyses, ezetimibe combination treatment was more effective in subjects with higher BMI, presence of T2DM, presence of sarcopenia, increased HOMA-IR, and increased baseline MRE.

Ezetimibe inhibits the absorption of dietary fat, including cholesterol, via NPC1-L1 [18]. Several previous animal models confirmed improvement in hepatic steatosis by ezetimibe. Improvement in triglyceride and free fatty acid by reduction of chylomicron synthesis in enterocytes [9], downregulation of SREBP-1c [19], or autophagy induction through AMPK activation and TFEB nuclear translocation [11] has been suggested as mechanisms by which ezetimibe contributes to improved hepatic steatosis and inflammation. However, previous studies on the effects of ezetimibe on hepatic steatosis in humans were inconsistent. Some showed that long-term treatment with ezetimibe improved histological indications of NAFLD (including steatosis) [20] whereas others, including Loomba et al., reported that while it reduced liver far by 15.3% compared to placebo, it produced no significant improvement in hepatic steatosis by MRI-PDFF [12].

Our study demonstrated that significant improvement in hepatic steatosis occurred after treatment with the ezetimibe combination, and there may be several reasons for this result. First, this study was conducted in combination with a statin. Unlike statins, which inhibit cholesterol synthesis in the liver, ezetimibe targets gastrointestinal cholesterol absorption within the small intestine. In this process, ezetimibe can inhibit an increase in dietary cholesterol absorption, which can arise from the use of statins [21]. NPC1-L1 inactivation causes multiple lipid transport defects and had a protective effect against diet-induced hyperlipidemia in a mouse model [22]. Dietary cholesterol exacerbates hepatic steatosis and inflammation in an LDL receptor-deficient mice model of metabolic syndrome [23]. In addition, hepatic NPC1-L1 induces hepatic steatosis by participating in cholesterol reabsorption from bile to the liver, which can be inhibited by ezetimibe [24]. This suggests that ezetimibe may have a greater effect on NAFLD in combination with a statin. Similarly, ezetimibe showed a prominent effect in the protection of cardiovascular diseases in a large randomized controlled trial (IMPROVE-IT study) in combination with a statin [13].

Second, differences in the characteristics of the current study population may have the potential to influence the ezetimibe effect. Subgroup analysis showed that combination treatment was more effective in improving hepatic steatosis in individuals with higher BMI, presence of T2DM or sarcopenia, higher HOMA-IR, and increased baseline MRE (≥2.1, the median value in this study). Inhibiting absorption of dietary cholesterol by ezetimibe can contribute to improvement in inflammation and insulin resistance [25]. Considering this mechanism, ezetimibe efficacy may be greater in subjects with obesity, higher insulin resistance, and T2DM, who have poor dietary habits and who may have elevated dietary cholesterol. Similarly, ezetimibe was more effective in protecting against cardiovascular disease in subjects with T2DM in our meta-analysis [26] and the results were consistent with a separate propensity score-matched cohort study [27]. The ability of ezetimibe to improve inflammation by modulating autophagy and inflammasomes [11] may also be greater in individuals with more advanced NASH or hepatic fibrosis. Therefore, a higher proportion of participants with insulin resistance in the presence of T2DM, which aggravates the inflammatory condition [28], could potentiate the effects of ezetimibe. Compared to the previous trial by Loomba et al. [12], our study treated all participants with the same dose of statin and recruited a higher proportion (>70%) of subjects with T2DM. These differences may be responsible for the apparently positive ezetimibe effect on NAFLD.

Although this study cannot fully explain its contributory mechanisms, there is evidence that ezetimibe changes the bile acid pool or microbiome [29]. Bile acid and microbiome are currently being studied as potentially important therapeutic targets for NASH or hepatic steatosis [30, 31]. The target protein of ezetimibe, NPC1-L1, known to be present in the intestine, was recently shown to be present in hepatocyte and Kupffer cells, affecting bile cholesterol reabsorption, which is expected to have a pleiotropic effect on hepatic steatosis [30, 32].

Progression from simple steatosis to NASH or fibrosis is of clinical importance. It is known that oxidative stress plays an important role in the progression of inflammation and fibrosis [33]. Ezetimibe has been shown to inhibit these actions through increased Nrf2 activity [34] or induction of autophagy flux [11]. In the markers related to inflammation that were evaluated in this study, only IL-18, an inflammasome-related cytokine, was significantly reduced in the ezetimibe combination group. IL-18 is involved in the inflammatory process and plays an important role in NAFLD [35]. This suggests that ezetimibe treatment may contribute to improvement in hepatic steatosis and the inflammatory process by reducing IL-18. Furthermore, fibrosis progression was evaluated by pre- and post-MRE and TE, and no significant improvement in fibrosis was found in either group. It may be that a 6-month treatment period is not long enough to show a difference. Considering the long progression from steatosis to fibrosis, further study is needed to understand the long-term effect of ezetimibe on hepatic fibrosis. On the other hand, there are clinical studies suggesting that statins are associated with a lower risk of hepatic fibrosis. Ciardullo et al. reported the lower odds of advanced fibrosis in statin users [36]. Statins may play an important role in NASH management in terms of reducing inflammation and oxidative stress [37]. Combination of statin and ezetimibe may contribute to the improvement of NAFLD by various mechanisms.

In this study, we also report improvement of hepatic steatosis in the monotherapy group. Previous studies of statins have shown inconsistent results regarding their potential contribution. Dongiovanni et al. reported the protective role of statins in individuals at risk of non-alcoholic steatohepatitis [38]. However, in a study by Nelson et al. [7], statin use was not associated with improvement in hepatic steatosis. In the current study, participants received counseling and education regarding healthy lifestyles and behavior modification. It is possible that this led to improvement of hepatic steatosis even in the statin alone group. A second possibility is that increased public interest in NAFLD contributed to better self-care by study participants. Since the study did not employ a placebo group, it is not possible to state definitively whether statin monotherapy improved hepatic steatosis. We observed significant weight loss in both study arms, and this may have accounted for some of the changes we observed in liver fat. All patients with NAFLD should receive lifestyle education; however, individual response to this may be different outside of a trial. Therefore, our results can be attenuated in the real-world setting. However, the main results of the current study that the combination of ezetimibe and rosuvastatin was significantly superior to rosuvastatin monotherapy in improving steatosis measured by MRI-PDFF suggest a beneficial role of ezetimibe in improving NAFLD.

There are several limitations in this study. Baseline hepatic steatosis was marginally lower in the monotherapy group compared to the combination group. Liver biopsy, the gold standard to measure hepatic steatosis or fibrosis, was not performed. In this study, it was confirmed that co-administration of ezetimibe with statin contributed to the improvement of hepatic steatosis. However, further evidence should be implemented to confirm clinically relevant effects of ezetimibe on hepatic fibrosis or clinical outcomes in NASH patients. The small sample size and short observation period of 6 months were not sufficient to determine whether concomitant use of ezetimibe could lead to improvement in hepatic fibrosis. We enrolled patients with hepatic steatosis documented by abdominal ultrasound, and one subject in each group excluded having no documented hepatic steatosis (<5% on MRI-PDFF) at baseline. We performed the final analysis as a per-protocol analysis, which may lead to an increased risk of selection bias. The open-label design implies that the interpretation of the results of this study should be made with caution. On balance, there are several notable strengths. First, we used the MRI-PDFF method, recognized as a highly reliable method of measuring hepatic steatosis [12], to evaluate the primary outcome. PDFF methods are reported to have higher diagnostic performance in noninvasive detection of hepatic steatosis in patients with NAFLD than CAP techniques [39], which might explain the discrepant findings regarding the degree of improvement in hepatic steatosis measured by PDFF or CAP. Second, we confirmed the effect of ezetimibe in combination with a statin, which is widely prescribed in the clinical environment. Third, we utilized MRE to confirm the effect on progression of hepatic fibrosis. We also measured by TE, another method to evaluate hepatic steatosis and fibrosis. Although the difference between groups was unclear, significant improvement in hepatic steatosis was confirmed only in the ezetimibe combination group. Fourth, in subgroup analyses, it was possible to identify characteristics of participants in whom ezetimibe acted more effectively on hepatic steatosis.

## Conclusions

In conclusion, the use of ezetimibe in combination with rosuvastatin significantly improved hepatic steatosis in patients with NAFLD. Individuals with higher BMI, T2DM, insulin resistance, and severe liver fibrosis were likely to be good responders to ezetimibe treatment. These data indicate that ezetimibe plus rosuvastatin is a safe and effective therapeutic option to treat patients with NAFLD and dyslipidemia.

## Supplementary Information

**Additional file 1: Appendix 1.** Detailed Exclusion criteria. **Appendix 2.** MRI-PDFF for fat quantification and MRE for liver fibrosis quantification. **Figure S1.** MRI map. **Table S1.** Pulse sequence parameters for magnetic resonance imaging. **Table S2.** Longitudinal Changes in Hepatic Steatosis and Fibrosis Using Fibroscan.

## Data Availability

The datasets generated during and/or analyzed during the current study are available from the corresponding author on reasonable request.

## Abbreviations

ALT: Alanine aminotransferase
AST: Aspartate aminotransferase
BMI: Body mass index
CAP: Controlled attenuation parameter
CRP: C-reactive protein
DM: Diabetes mellitus
ESSENTIAL: Effects of Statin Monotherapy and Statin/Ezetimibe Combination Therapy on Non-alcoholic Steatohepatitis in patients with Hyperlipidemia and Fatty Liver
HDL-C: High-density lipoprotein cholesterol
HOMA-IR: Homeostatic model assessment insulin resistance
IL: Interleukin
LDL-C: Low-density lipoprotein cholesterol
LSM: Liver stiffness measurement
MRE: Magnetic resonance elastography
MRI-PDFF: Magnetic resonance imaging-derived proton density fat fraction
NAFLD: Non-alcoholic fatty liver disease
NASH: Non-alcoholic steatohepatitis
NPC-L1: Niemann-Pick C1-like 1
ROI: Regions of interest
SMI: Skeletal muscle mass index
SREBP-1c: Sterol regulatory element-binding protein-1c
T2DM: Type 2 diabetes
TE: Transient elastography
WC: Waist circumference
