# Supplementary material for: Ezetimibe combination therapy with statin for non-alcoholic fatty liver disease: an open-label randomized controlled trial (ESSENTIAL study)
Source: BMC Med. 2022 Mar 21;20:93. doi: 10.1186/s12916-022-02288-2 (PMC8935785; doi:10.1186/s12916-022-02288-2)
Supplement: Supplementary file 1 — Additional file 1: Appendix 1. Detailed Exclusion criteria. Appendix 2. MRI-PDFF for fat quantification and MRE for liver fibrosis quantification. Figure S1. MRI map. Table S1. Pulse sequence parameters for magnetic resonance imaging. Table S2. Longitudinal Changes in Hepatic Steatosis and Fibrosis Using Fibroscan. [file 12916_2022_2288_MOESM1_ESM.docx]

**Supplementary Appendix.**

1. **Detailed Exclusion criteria**

Patients were excluded if they met any of the following exclusion criteria:

Diabetes other than type 2 diabetes, including type 1 diabetes and gestational diabetes; previous history of ezetimibe treatment or discontinuation due to side effects; history of discontinuation of statin therapy due to side effects; uncontrolled diabetes (HbA1c >9.0%); acute or chronic metabolic acidosis, including diabetic ketoacidosis with or without coma or a history of ketoacidosis within 6 months of enrollment; use of thiazolidinedione and sodium-glucose cotransporter 2 inhibitor drugs (which can affect hepatic steatosis); alcoholic fatty liver disease in the last 2 years (210 g/week for men, 140 g/week for women); chronic liver disease including hemochromatosis, liver cancer, autoimmune liver disease, Child-Pugh score >7; platelets <75,000 mm^2^, prothrombin time >16s, viral hepatitis -A, B; current medication that can cause fatty liver (amiodarone, methotrexate, tamoxifen, valproate, corticosteroids, etc.); treatment with oral or parenteral corticosteroids for 14 consecutive days within 8 weeks of enrollment; genetic conditions including galactose intolerance, Lapp lactose dehydrogenase deficiency, or glucose-galactose uptake disorder; malnutrition, starvation, weakness (including severe infection, pre- or post-operative trauma), pituitary dysfunction, or adrenal insufficiency; serum levels of alanine aminotransferase, aspartate aminotransferase or alkaline phosphatase >5-fold elevation in upper limit normal (ULN) range or 5-fold elevation in serum total bilirubin level; current medication for weight loss; history of malignant tumor/s within the past 2 years, current treatment of malignant tumor/s, or tumor progression; history of substance abuse or alcohol intoxication within 12 weeks; human immunodeficiency virus (HIV) infection; acute cardiovascular disease within 12 weeks including unstable angina, myocardial infarction, transient ischemic attack, cerebrovascular disease, coronary artery bypass grafting, or coronary intervention; renal failure, chronic renal disease (estimated glomerular filtration rate <60 mL/min/1.73 m^2^); anemia (hemoglobin <10.5 g/dl); surgical or medical condition that may affect the absorption, distribution, metabolism and excretion of a drug, including but not limited to gastrectomy, gastroenterostomy; history of major gastrointestinal surgery including small bowel resection, gastrointestinal bypass, gastrointestinal stapling, current active gastritis, gastrointestinal/rectal bleeding, active inflammatory bowel syndrome within the last 12 months; pregnant or lactating women.

1. **MRI-PDFF for fat quantification and MRE for liver fibrosis quantification**

MRI was performed using a 3.0-T system (Ingenia CX, Philips Medical Systems, Best, Netherlands) at baseline and after 24 weeks of study participation. For hepatic fat quantification, MRI-PDFF sequence (mDIXON Quant) was used, and a fat fraction map was automatically generated by the manufacturer’s console. For liver fibrosis measurement, MRE was performed using a 2-dimensional gradient-echo sequence and a passive driver placed on the participant’s right upper abdomen. The detailed pulse sequence parameters for MRI-PDFF and MRE sequences are displayed in a supplementary table 1. All images were evaluated by one abdominal radiologist (H.R.) who was blinded to treatment assignment. For the fat fraction measurement, three ROIs of 300-400 mm^2^ were identified in each of the nine liver segments on the fat fraction map, avoiding blood vessels, bile ducts, and focal hepatic lesions. The average of total 27 PDFF measurements was used as the liver fat fraction. In pre- and post-treatment MRIs, ROIs were placed at the same location as possible. For liver fibrosis measurement, ROIs were drawn in four slices of the stiffness map to include as much liver parenchyma as possible, avoiding blood vessels, bile ducts, focal hepatic lesions and the subcapsular area, and the average of all measurements was used.

**Supplementary figure 1**

**
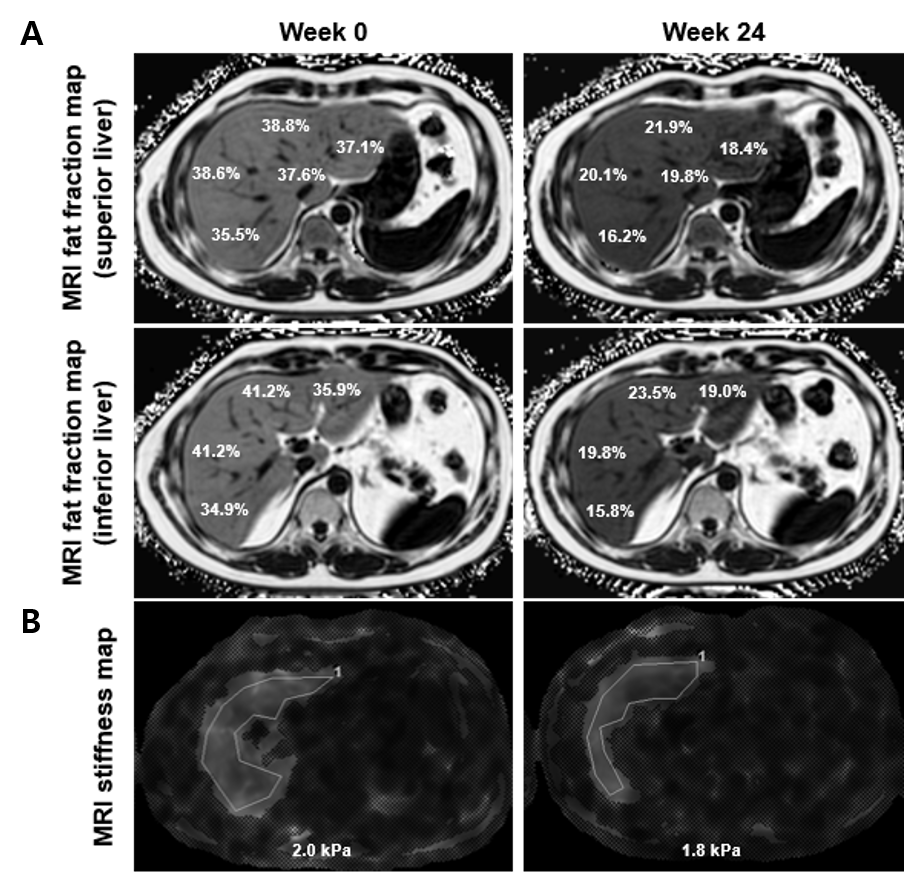
**

(A) Whole-liver fat mapping with MRI-PDFF for a single patient.

MRI-PDFF measurements of liver segments 1, 2, 4a, 7, and 8 in the superior plane (upper panel) and of liver segments 3, 4b, 5, and 6 in the inferior plane (lower panel) are shown at weeks 0 (left column) and 24 (right column). Three regions of interest (ROIs) of 300–400 mm^2^ were placed in each of the nine liver segments on a fat fraction map, avoiding vessels, bile ducts, and focal hepatic lesions. The average of 27 total PDFF measurements was used as liver fat fraction. In before- and after-treatment MRIs, ROIs were placed at the same location as possible.

(B) Liver stiffness measured by MRE using 2-dimensional gradient-echo sequence and a passive driver placed on patient’s right upper abdomen.

**Supplementary Table 1. Pulse sequence parameters for magnetic resonance imaging**

| **Parameter** | **Value** |
| --- | --- |
| **Proton density fat fraction** |  |
| Technique | mDIXON Quant |
| Pulse sequence | 3-dimensional gradient echo |
| Field of view (cm) | 40 |
| Matrix | 160ⅹ160 |
| Section thickness (mm) | 6 |
| Slice spacing (mm) | 3 |
| Repetition time (msec) | 5.82 |
| First echo time (msec) | 1 |
| Echo time spacing (msec) | 0.7 |
| No. of echo trains/no. of echoes | 1/6 |
| Bandwidth (approximate kHz/pixel) | 2.6 |
| Flip angle (degrees) | 3 |
| Readout type | Bipolar |
| **Elastography** |  |
| Pulse sequence | 2-dimensional gradient echo |
| Field of view (cm) | 40 |
| Matrix | 304ⅹ89 |
| No. of signals acquired | 1 |
| Echo time (msec) | 20.59 |
| Repetition time (msec) | 50 |
| Bandwidth (kHz/pixel) | 0.25 |
| No. of sections | 4 |
| Section thickness (mm) | 10 |
| Slice spacing (mm) | 11 |
| No. of phases | 4 |
| MEG frequency (Hz) | 60 |
| Axis of MEG | z |
| Driver frequency (Hz) | 60 |
| Driver cycles per trigger | 3 |
| No. of breath holds | 4 |
| Acceleration | SENSE |
| Acceleration factor | 2 |
| Imaging time | 18 sec.ⅹ4 |

**Supplementary Table 2.** Ezetimibe plus Rosuvastatin versus Rosuvastatin Monotherapy: Longitudinal Changes in Hepatic Steatosis and Fibrosis Using Fibroscan

|  | **Ezetimibe + Rosuvastatin (n=31)** | | |  | **Rosuvastatin Alone (n=33)** | | | **Difference between groups** |
| --- | --- | --- | --- | --- | --- | --- | --- | --- |
|  | Baseline | Post-treatment | p-value |  | Baseline | Post-treatment | p-value | p-value |
| CAP, dB/m | 321.0 (46.0) | 287.0 (46.0) | 0.018 |  | 323.0 (34.5) | 311.0 (60.5) | 0.104 | 0.253 |
| LSM, kPa | 6.0 (3.2) | 6.1 (3.0) | 0.339 |  | 6.7 (2.6) | 6.0 (3.8) | 0.881 | 0.623 |

Data are expressed as means (SD) or difference with p-values from paired t-test or intention-to-treat analysis.

Abbreviations: CAP, controlled attenuation parameter; LSM, liver stiffness measurement.
